# Synthetic DNA Vaccines Adjuvanted with pIL-33 Drive Liver-Localized T Cells and Provide Protection from *Plasmodium* Challenge in a Mouse Model

**DOI:** 10.3390/vaccines8010021

**Published:** 2020-01-10

**Authors:** Sophia M. Reeder, Emma L. Reuschel, Mamadou A. Bah, Kun Yun, Nicholas J. Tursi, Kevin Y. Kim, Jacqueline Chu, Faraz I. Zaidi, Ilknur Yilmaz, Robert J. Hart, Benjamin Perrin, Ziyang Xu, Laurent Humeau, David B. Weiner, Ahmed S. I. Aly

**Affiliations:** 1 The Vaccine Center, Wistar Institute, Philadelphia, PA 19104, USA; 2 Perelman School of Medicine, University of Pennsylvania, Philadelphia, PA 19104, USA; 3 Beykoz Institute of Life Sciences and Biotechnology, Bezmialem Vakif University, Istanbul 34820, Turkey; 4 Department of Tropical Medicine, Tulane University, New Orleans, LA 70112, USA; 5 Inovio Pharmaceuticals, Inc., Plymouth Meeting, PA 19462, USA

**Keywords:** malaria, *Plasmodium*, liver stage, exported proteins, DNA vaccines

## Abstract

The need for a malaria vaccine is indisputable. A single vaccine for *Plasmodium* pre-erythrocytic stages targeting the major sporozoite antigen circumsporozoite protein (CSP) has had partial success. Additionally, CD8+ T cells targeting liver-stage (LS) antigens induced by live attenuated sporozoite vaccines were associated with protection in human challenge experiments. To further evaluate protection mediated by LS antigens, we focused on exported pre-erythrocytic proteins (exported protein 1 (EXP1), profilin (PFN), exported protein 2 (EXP2), inhibitor of cysteine proteases (ICP), transmembrane protein 21 (TMP21), and upregulated in infective sporozoites-3 (UIS3)) expressed in all *Plasmodium* species and designed optimized, synthetic DNA (synDNA) immunogens. SynDNA antigen cocktails were tested with and without the molecular adjuvant plasmid IL-33. Immunized animals developed robust T cell responses including induction of antigen-specific liver-localized CD8+ T cells, which were enhanced by the co-delivery of plasmid IL-33. In total, 100% of mice in adjuvanted groups and 71%–88% in non-adjuvanted groups were protected from blood-stage disease following *Plasmodium yoelii* sporozoite challenge. This study supports the potential of synDNA LS antigens as vaccine components for malaria parasite infection.

## 1. Introduction

Malaria continues to cause disease in 290 million humans annually, with a death toll of approximately 435,000 per year. Malaria infections are caused by the *Plasmodia* parasite, which has a complex life cycle, with stages in both vertebrate and invertebrate hosts. Five species of *Plasmodium* are infectious to humans: *Plasmodium falciparum*, *Plasmodium vivax*, *Plasmodium malariae*, *Plasmodium ovale*, and *Plasmodium knowlesi*. While *P. falciparum* is responsible for the majority of deaths caused by malaria, *P. vivax* has recently been shown to be the cause of approximately 25% of severe malaria in Southeast Asia, and multi-drug resistant *P. vivax* has been identified [1]. Infection in humans begins when the human is bitten by an infected female Anopheles mosquito, which inoculates sporozoites in the dermis. Sporozoites subsequently travel through the blood to invade hepatocytes. The liver-stage (LS) parasite is separated from the infected hepatocyte by a selective parasitophorous vacuolar membrane (PVM) of host hepatocyte plasma membrane origin [2,3]. The growing LS parasite acquires nutrients from its host hepatocyte and at the same time prevents its apoptosis [4,5,6,7]. Each infected hepatocyte can generate tens of thousands of merozoites, which will subsequently rupture from the liver and start the pathogenic blood stage of infection.

The main sporozoite antigen that covers the entire sporozoite surface is circumsporozoite protein (CSP) [2]. CSP was and still is a main target for vaccination trials over the last several decades mainly because of the antigenicity of its domains and the sporozoite neutralizing effect of CSP-antibodies *in vitro* and *in vivo* [8,9]. The most advanced subunit malaria vaccine is RTS,S, which is based on repeat regions of CSP coupled with Hepatitis B envelope protein and the potent AS01 adjuvant. RTS,S induces impressive CSP antibody responses, resulting in 46% vaccine efficacy against clinical malaria in children and 27% efficacy in infants in the 18 months following immunization [10]. The antibody responses significantly wane over time, as does protection [10,11,12,13].

The only vaccination method that has led to long-lasting complete sterile protection against malaria parasite challenges in animals and in controlled human malaria infection (CHMI) is immunization with live irradiation-attenuated sporozoites [14,15]. Field studies in Burkina Faso, Mali, Kenya, Gabon, and Tanzania are currently following up on this approach [16]. While these studies are important for the efforts to develop an effective malaria vaccine, attenuation by irradiation is not easily standardized for human use. Over-irradiated sporozoites confer little protection while under-irradiation provides risk for breakthrough infections. More recently, attenuation of sporozoites was conducted by targeted deletion of genes that encode LS essential proteins in the mouse model. In both attenuation models, *Plasmodium* sporozoites invade hepatocytes within vacuoles, then cease growth completely and do not cause *Plasmodium* infection of the blood [2,3]. The protection conferred by attenuated sporozoites was confirmed to be mainly mediated by CD8+ T cells targeting LS antigens and not by antigens presented on the surface of migrating sporozoites [17,18,19,20,21]. Recent studies pointed out that when compared to attenuated strains that cease their LS development early, attenuated strains that grow longer in hepatocytes before ceasing growth led to more significant protective immune responses [17]. This indicated that significant exposure to LS antigens can enhance vaccine effectiveness. Further, liver-associated T cells have been implicated in anti-malarial immunity following irradiated sporozoite vaccination [22,23]. When T cells lack CXCR6, a cell surface marker highly expressed by liver-infiltrating CD8 T cells, there is a reduction of liver-associated memory and sporozoite immunity [22]. Very recent studies (including this report) support that CD8 tissue resident memory T cells appear important for targeting of LS malaria following vaccination [23,24,25]. It appears that LS antigens represent important candidates for inducing protective CD8+ T cell responses in the attenuated sporozoite model. 

Despite the evident potential of live attenuated parasites as vaccines, the feasibility and large-scale application of live attenuated sporozoites that have to be produced aseptically in mosquitoes in high amounts is still in development [8]. Alternative vaccine platforms remain of interest. 

We hypothesized that a vaccine comprised of multiple LS antigens delivered as optimized synthetic DNA plasmid cassettes might induce protective immunity similar to that observed with live attenuated parasite models. We focused on the following antigens, which are expressed across *Plasmodia*: EXP1, PFN, EXP2, ICP, TMP21, and UIS3. EXP1 (exported protein 1) is a glutathione transferase, located at the parasite-host interface, which efficiently degrades cytotoxic hematin, and is associated with the metabolism of and susceptibility to artesunate, a frontline anti-malarial drug [26,27]. Additionally, it has been shown that EXP1 contains degenerate T cell epitopes [28]. EXP1 has been previously shown to be immunogenic in a mouse model of malaria [29]. There is evidence in humans that EXP1 may be an important anti-malarial target, as a positive antibody response to EXP1 correlated with a statistically significant decrease in malarial infection in children in Burkina Faso [30]. PFN (profilin) has been detected in all life cycle stages, including sporozoites and merozoites, and abundant PFN expression suggests PFN is important for *Plasmodium* life cycle progression [31,32]. Like all apicomplexans, *Plasmodium* utilizes a highly specialized microfilament system for motility and host cell invasion, and profilin plays a key role as an actin-sequestering protein [33,34]. It has been shown that disturbing expression of PFN results in complete life cycle arrest [35]. Similarly to EXP1, EXP2 (exported protein 2) is an integral vacuolar protein [36]. EXP2 resides primarily on the vacuolar face of the PVM, and likely constitutes the membrane pore [37]. In *Plasmodium*, ICP (inhibitor of cysteine proteases) has been shown to be necessary for malaria transmission from mosquitos to mammals, sporozoite motility, erythrocyte invasion [38], and liver-stage development [39]. While little is known about TMP21 (transmembrane protein 21), vaccination with TMP21 reduced liver-stage parasite load in a mouse model and has been shown to contribute to the protective immunity elicited by whole parasite vaccinations [40]. The antigen UIS3 (upregulated in infective sporozoites-3) is a membrane protein that is localized to the PVM in infected hepatocytes [41] and interacts directly with host liver-fatty acid binding protein (L-FABP) [42]. UIS3 is essential for early liver-stage development [43] and vaccination with a ChAd63-MAV vaccine containing UIS3 has been shown to be partially protective in a mouse model [44]. 

One of the strengths of the synthetic DNA vaccine platform is the ease with which antigens can be co-formulated with each other and with molecular adjuvants in the clinic [45,46,47,48]. Given the focus on induction of cellular immunity, we explored using synthetic DNA-encoded plasmid IL-33 (pIL-33) as a unique molecular adjuvant. pIL-33 is a member of the IL-1 family, which although originally associated with Th2 immunity, has also been reported to facilitate the generation of protective Th1 and CD8 T cell immunity [46]. pIL-33 has immunoadjuvant effects in an HPV-associated model for cancer immunotherapy in which cell-mediated immunity is critical for protection and has been reported to enhance potent antigen-specific effector and memory T-cell immunity in a synthetic DNA vaccine setting [48]. The ability to induce potent cell-mediated immunity is important for a vaccine against LS malaria, as is the fact that IL-33 is predominantly expressed at the epithelial barrier as the first line of defense against pathogenic threats, activating a variety of important immune cells [49], which may be relevant to the necessity for *Plasmodium* sporozoites to transverse through the dermis before LS infection. Consequently, IL-33 was chosen as a molecular adjuvant for this study. Another strength of the DNA vaccine platform is its ability to drive functional, localized cell-mediated immunity (CMI) in the clinic [47,50]. Prior work studying the immunity from a hepatitis B DNA vaccine showed the ability of this platform to drive vaccine-specific CTLs to traffic to the liver, an organ that is known to be tolerogenic and suppress T cell responses [51]. The ability of this platform to drive vaccine-specific CTLs in this location highlights the potential to drive relevant, functional CMI in this context. 

## 2. Materials and Methods

### 2.1. Construct Design

**Figure 1:**
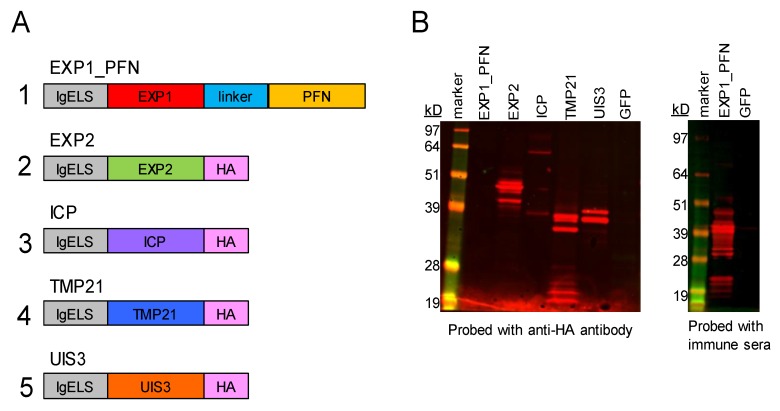
DNA vaccine construct design and *in vitro* expression. (**A**) Schematic diagram of *Plasmodium yoelii* (*Py*) gene inserts used to generate the codon-optimized DNA vaccine constructs. The schematic details leader sequence (IgE), gene insert, and presence or absence of HA tag. All constructs except for EXP1_PFN (exported protein 1_profilin) contain an HA tag. (**B**) Expression of *Py* proteins detected by SDS-polyacrylamide gel electrophoresis and western blot of lysate from transfected 293T cells. Protein expression was detected by probing for the HA tag when present with an anti-HA antibody, or with immune sera from immunized mice for the EXP1_PFN plasmid. GFP is included as a negative control.

Protein sequences for selected *Plasmodium* antigens were accessed on PlasmoDB, the *Plasmodium* genomics resource (EXP1: PY17X_0928700, PFN: PY17X_0836400, EXP2: PY17X_1339000, ICP: PY17X_0816300, TMP21: PY06414, UIS3: PY17X_1402400). While all antigens selected are expressed across *Plasmodia*, the vaccine constructs were matched to *Plasmodium yoelii* 17X (*Py*), to reflect the planned challenge model, which was *P. yoelii* 17X-NL (non-lethal) strain. The synthetic DNA vaccine constructs were codon optimized for mice and humans and were incorporated into a modified pVax vector with an IgE leader sequence and an HA tag where indicated in Figure 1. 

### 2.2. Western Blot

In order to assess in vitro expression of the vaccine constructs, 293T cells (ATCC^®^ CRL-3216™) were plated in 6-well plates at 0.5–0.7 × 10^6^ cells per well in 2 mL DMEM (Gibco) + 10% FBS and incubated overnight at 37 °C in 5% CO_2_. Cells were transfected with 5 μg DNA using TurboFectin 8.0 transfection kit and incubated for 48 h. After transfection, supernatants and lysates were collected for western blot analysis. Samples were prepared with NuPAGE LDS Sample Buffer, 10× Reducing Agent, and deionized water. Sample mixture was incubated at 70 °C for 10 min. A volume of 25 μL of sample was loaded per well onto an Invitrogen NuPAGE Bis-Tris Gel in 1 × NuPage MOPS running buffer. The gel was run at 150 V for 50 min. Protein was transferred to a methanol activated PVDF membrane using the iBlot 2 Dry Blotting System (Life Technologies, Carlsbad, CA, USA). The membrane was blocked for 1 h at room temperature with Li-Cor Odyssey Blocking Buffer followed by overnight incubation at 4 °C with primary antibody. Primary antibody was either an anti-HA antibody (ThermoFisher, Waltham, MA, USA) to detect expression of the constructs which contain an HA tag, or post-immune sera collected from immunized mice to detect expression of the EXP1_PFN construct which did not have an HA tag. After washing, the membrane was incubated for 1 h at room temperature with IRDye labeled secondary antibody (Li-Cor) and then imaged using an Odyssey CLx imager.

### 2.3. Immunization and CELLECTRA Electroporation

Female BALB/cJ mice were ordered from the Jackson Laboratory aged 6–8 weeks and were housed in the Wistar Institute Animal Facility. All animal handling was conducted according to the approved protocols of the Institutional Animal Care and Use Committee (IACUC) of the Wistar Institute (Protocol 201153). Mice were immunized with 30 μg DNA vaccine construct with or without 25 μg of plasmid pIL-33 delivered intramuscularly using the CELLECTRA 3P adaptive constant current electroporator [52]. When mice were immunized with a cocktail of antigens, 30 μg of each DNA vaccine construct was used. Depending on the experiment, mice were immunized 4 or 3 times at 3 week intervals. Blood was collected 1 week post each vaccination for sera isolation. One week post final immunization mice were euthanized and splenocytes and hepatocytes were collected for immune analysis. 

### 2.4. Immune Cell Isolation

After euthanasia, liver and spleen tissue were removed for immune cell isolation. Before liver excision, the hepatic portal vein exiting the liver was cut and 5–10 mL of cold PBS solution was administered through the left ventricle to profuse the liver until blanched. Once perfused the liver was removed and placed in cold complete media (DMEM + 10% FBS + 20 mM HEPES + 1 × Pen/Strep). Livers were quickly homogenized for 1 min at normal speed using a Stomacher 80 (Seward). Homogenates were transferred to 6-well plates with 10 mL of digest media (DMEM + 20 mM HEPES + 0.1 mg/mL Collagenase 4 + 0.02 mg/mL DNase) added and plates were incubated at 37 °C for 30 min. Cells were filtered through a 100 μm mesh strainer, rinsed with PBS, and then centrifuged at 300 rpm for 1 min. The supernatant containing hepatocytes was transferred to a new tube and spun down for 5 min at 1500 rpm. The cell pellet was resuspended in 40% Percoll (Sigma-Aldrich, St. Loius, MO, USA), underlaid with 80% Percoll, and spun at 3000 rpm for 20 min with slow acceleration and deceleration. Hepatocytes at the interface of the two Percoll layers were removed and diluted in complete media, then counted using a COUNTESS II (Invitrogen Carlsbad, CA, USA) and trypan blue (Gibco).

Spleens were removed and placed in R10 (RPMI + 10% FBS + 1 × Pen/Strep). Spleens were homogenized for 1 min on high using a Stomacher 80. The cell solution was filtered through a 100 μm strainer and spun down for 10 min at 1200 rpm. Cells were resuspended in 5 mL ACK lysis buffer (Gibco) and incubated for no more than 5 min. After washing with PBS, cells were spun for 10 min at 1200 rpm. Splenocytes were then resuspended in 20 mL of R10 and counted.

### 2.5. ELISPOT

Mouse IFN-γ ELISpot PLUS (Mabtech, Cincinnati, OH, USA) plates were used as directed. Briefly, plates were washed with PBS and blocked with R10 for 30 min. Wells were seeded in triplicate with 200,000 cells in 100 μL R10. Cells were stimulated with peptide pools of 15mers overlapping by 11 amino acids spanning the entire vaccine antigen at a final concentration of 5 μg/mL per peptide. R10 and Concanavalin A were used as negative and positive controls, respectively. Plates were incubated for 18 h at 37 °C with 5% CO_2_. Plates were developed as directed, scanned, and counted using a CTL ImmunoSpot S6 Universal Analyzer. Data was exported to Microsoft Excel and GraphPad Prism 8 for analysis. 

### 2.6. Flow Cytometry

Wells were seeded with 1,000,000 cells in 100 μL of R10. Cells were stimulated with peptides at a final concentration of 5 μg/mL per peptide in the presence of Protein Transport Inhibitor (eBioscience, San Diego, CA, USA). R10 and Cell Stimulation Cocktail (eBioscience, San Diego, CA, USA) were used as negative and positive controls, respectively. Plates were incubated for 6 h at 37 °C with 5% CO_2_. Following incubation, cells were washed with PBS, and stained with Live/Dead fixable aqua dead cell stain kit (ThermoFisher, Waltham, MA, USA) in PBS. Cells were then stained for extracellular markers in FACS buffer (1% FBS in PBS), fixed and permeabilized with BD Fix/Perm, stained for intracellular markers and cytokines in BD perm/wash, resuspended in FACS buffer, and run on BD LSRII flow cytometer (BD Biosciences, Franklin Lake, NJ, USA). Splenocytes and hepatocytes were stained with following panel: LiveDead Aqua (Invitrogen, L34957, Carlsbad, CA, USA), CD19-V450 (BDHorizon, 560375, Franklin Lakes, NJ, USA), CD3-AF700 (BioLegend, 100216, San Diego, CA, USA), CD4-FITC (BD Pharmingen, 553047, Franklin Lakes, NJ, USA), CD8-BV605 (BioLegend, 100744, San Diego, CA, USA), IFNγ-APC (BioLegend, 505810, San Diego, CA, USA), TNFα-PE (eBioscience, 12-7321-82, San Diego, CA, USA), IL-2-PE-Cy7 (eBioscience, 25-7021-82, San Diego, CA, USA), and CXCR6-BV421 (Biolegend, 151109, San Diego, CA, USA). Gates were set using FMOs for each stain. Data were exported and analyzed in GraphPad Prism 8.1.1.

### 2.7. Immunofluorescence

Hepa1–6 cells (a cell line derived from mouse liver cells, ATCC^®^ CRL-1830™) were plated on pre-coated Poly-D-Lysine (Corning) 8 chambered wells at 100,000 cells per well in 400 μL DMEM (Gibco) + 10% FBS and incubated overnight at 37 °C in 5% CO_2_. Cells were transfected with 1 μg DNA using Thermofisher Lipofectamine 3000 transfection kit and incubated for 48 h. Media was removed, and cells were washed twice with PBS (Gibco) for 5 min and then fixed with 2% PFA in PBS for 5 min. Cells were again washed twice with PBS followed by blocking in 5% goat serum + 0.05% Tween-20 for 1 h at room temperature followed by two 5 min PBS washes. Cells were incubated with 500 μL pooled mouse sera at a 1:50 dilution in dilution buffer (1% BSA + 0.05% Tween-20 in PBS) at room temp for 1 h. After washing three times with PBST (0.05% Tween-20 in PBS) for 5 min each, cells were incubated with 300 μL of anti-IgG-AF488 secondary antibody diluted in dilution buffer at room temp for 1 h. Cells were washed with PBST for 5 min, then incubated with DAPI (Hoechst 33,342 Fluorescent Stain, Thermo Scientific, Waltham, MA, USA) at a 1:5000 dilution in PBST for 5 min, followed by a final wash with PBST. Cells were imaged using a Leica TCS SP5 confocal microscope. 

### 2.8. Mosquito Feeding and Sporozoite Extraction

Six-to-eight-week-old female Swiss Webster (SW) mice (purchased from Envigo, Indianapolis, IN, USA) were used for mosquito feeding experiments to generate salivary gland sporozoites for challenge studies. All animal handling was conducted according to the approved protocols of the Institutional Animal Care and Use Committee (IACUC) of Tulane University (Protocol 4258R). Mosquito feeding experiments were conducted with *P. yoelii* 17X-NL wild-type parasites as previously described [53,54,55]. Briefly, SW mice, treated with phenylhydrazine, are injected intravenously with 1 million blood stage parasites, and on day 3 post infection an exflagellation assay is used to confirm the availability and formation of *P. yoelii* male microgametes. Mosquito feeding is conducted by allowing about 150 female mosquitoes to feed on a mouse anesthetized with ketamine/xylazine for 15 min. Salivary gland sporozoite extraction was conducted by dissection of the salivary glands of infected female mosquitoes at day 14 or 15 post mosquito feeding (pmf) in RPMI incomplete medium, as previously described [17,56,57]. Collected salivary glands were mechanically disrupted with a pestle and the salivary gland sporozoites were counted using a hemocytometer. Doses of 250 sporozoites in 150 µL incomplete RPMI were prepared as previously described [22,23,26]. 

### 2.9. Challenge Study

Six-to-eight-week-old female BALB/cJ mice (purchased from Jackson Laboratory, Bar Harbor, ME) were used for challenge experiments. Mice were put under a red heating lamp 5–10 min before injection. Each dose of 250 sporozoites was loaded in 27G insulin syringes and was injected intravenously in the tail vein of immunized mice. Giemsa-stained thin blood smears were checked every day (at least 50 whole microscopy fields at 1000×) for blood stage parasites, starting from day 3 until day 10 post infection [17,56,57]. 

## 3. Results

### 3.1. synDNA Vaccine Construct Design and In Vitro Expression

Previously identified LS proteins were optimized and encoded into a modified pVax plasmid. Plasmid 1 contains an IgE leader sequence, the *EXP1* gene sequence, a linker sequence, and the *PFN* gene sequence. Plasmids 2, 3, 4 and 5 each contain an IgE leader sequence, the gene sequence for *EXP2*, *ICP*, *TMP21*, or *UIS3* respectively, and an HA tag (Figure 1A). While all LS proteins selected are expressed across *Plasmodia*, the synthetic optimized DNA vaccine constructs were matched for *Plasmodium yoelii* (*Py*), to reflect the planned challenge model. 

*In vitro* expression of *Py* constructs in transfected 293T lysates was detected by western blot. Expression was confirmed for plasmids 2–5 by detection of the HA tag. Expression for plasmid 1, which lacked an HA tag, was confirmed by probing with post-immune sera from mice immunized with the construct. GFP transfection was used as a negative control (Figure 1B). 

### 3.2. Py LS Vaccine Constructs Delivered Individually Elicit a Robust and Polyfunctional T Cell Response

**Figure 2:**
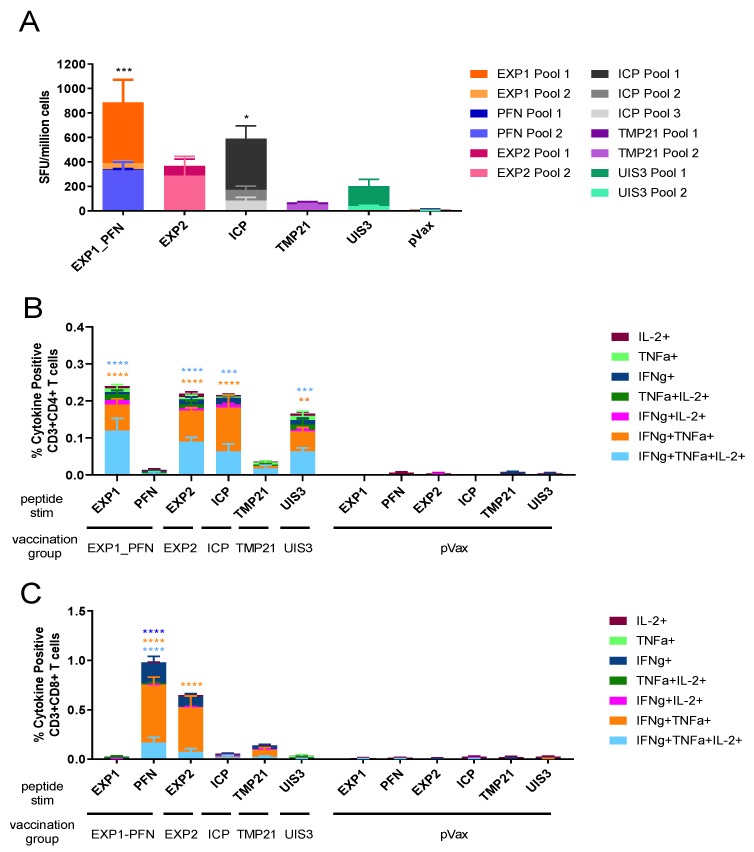
Functional profile of cellular immune responses elicited by individual *Py* DNA vaccines in mice. Mice were immunized 4 times at 3-week intervals with the indicated constructs. Splenocytes were collected 1 week after the final immunization. (**A**) The *Py* antigen-specific cellular immune response measured by IFNγ ELISPOT of splenocytes 1 week after final immunization with the indicated vaccine. Cells were stimulated for 18 h with peptide pools encompassing the entire protein. A one-way ANOVA with Dunnet’s multiple comparison test was used to compare each vaccine group to the pVax control group. (**B**,**C**) The *Py* antigen-specific cytokine production profile of CD4+ (**B**) and CD8+ (**C**) T cells from spleens 1 week after final immunization with the indicated vaccine. Cells were stimulated with pooled peptides for 6 h, stained for intracellular production of IFNγ, TNFα, and IL-2, and then analyzed by flow cytometry. The bar graph shows subpopulations of mono-, double-, and triple-positive CD4+ and CD8+ T cells. A two-way ANOVA with Tukey’s multiple comparisons test was used to compare cytokine production between each vaccine group and the pVax control group. Asterix color indicates which cytokines were significantly different between vaccine and control. * = *p* < 0.05, ** *p* < 0.01, *** = *p* < 0.001, and **** = *p* < 0.0001. Values represent the mean responses in each group (*n* = 5) ± SEM.

To assess the cellular immune response to LS antigen vaccination, groups of five mice were immunized 4 times at 3-week intervals with one of the five constructs individually, or the empty vector pVax. One week after final immunization splenocytes were collected for immune analysis and antigen-specific cytokine production was assessed by IFNγ ELISPOT and flow cytometry. All five constructs induced detectable IFNγ cellular responses, with EXP1_PFN and ICP being the highest inducers. EXP2 and UIS3 induced lower, but still robust levels of IFNγ secreting cells, and TMP21 induced readily detectable, but the lowest, levels of IFNγ secreting cells (Figure 2A). The functional profile of antigen-specific CD4+ (Figure 2B) and CD8+ (Figure 2C) T cells was analyzed by flow cytometry. Mono-, double-, and triple-positive CD4+ and CD8+ T cells releasing the cytokines IFNγ, TNFα, and IL-2 are shown. CD4+ T cells responded most highly to EXP1, EXP2, ICP, and UIS3. In contrast, CD8+ T cells responded most highly to PFN and EXP2. As expected, ELISPOT and flow cytometry responses in mice immunized with the empty vector control, pVax, were negligible (Figure 2).

### 3.3. Co-Formulated Py LS Vaccines Delivered with and without Plasmid IL-33 Adjuvant Elicit a Robust and Polyfunctional T Cell Response

**Figure 3:**
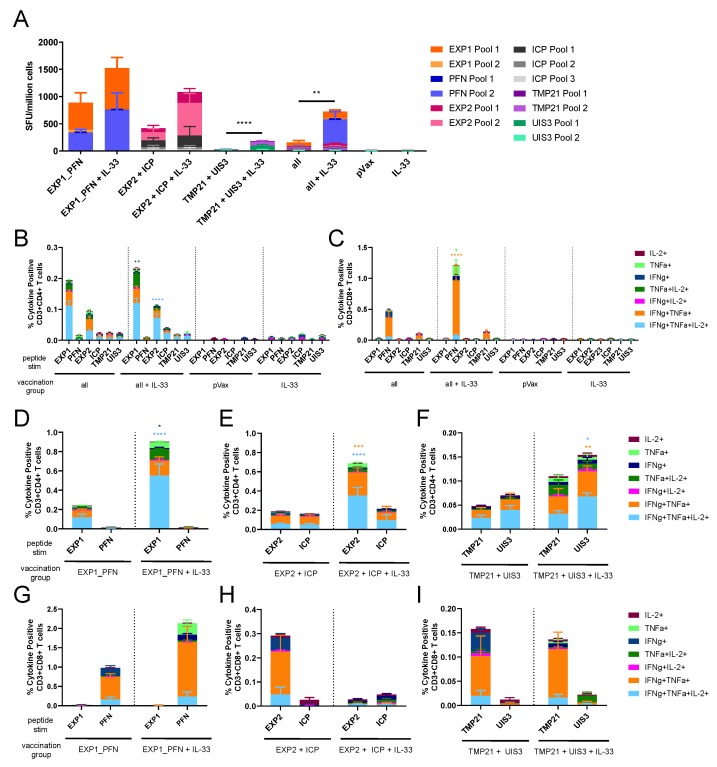
Functional profile of cellular immune responses elicited by co-formulated *Py* DNA vaccines in mice. Mice were immunized 4 times at 3-week intervals with the indicated co-formulation of vaccine constructs with and without plasmid IL-33. Splenocytes were collected 1 week after the final immunization. Immunization groups were: All vaccine constructs with or without IL-33, EXP1_PFN (exported protein 1_profilin) with or without IL-33, EXP2 (exported protein 2) and ICP (inhibitor of cysteine proteases) with or without IL-33, and TMP21 (transmembrane protein 21) and UIS3 (upregulated in infective sporozoites-3) with or without IL-33. (**A**) The *Py* antigen-specific cellular immune response induced by the indicated *Py* DNA vaccine co-formulation measured by IFNγ ELISPOT. Cells were stimulated for 18 h with peptide pools encompassing the entire protein. T-tests were used to compare groups with and without IL-33. (**B**–**I**) The *Py* antigen-specific cytokine production profile of CD4+ (**D**–**F**) and CD8+ (**G**–**I**) T cells induced by the indicated *Py* DNA vaccine co-formulation. Cells were stimulated with pooled peptides for 6 h, stained for intracellular production of IFNγ, TNFα, and IL-2, and then analyzed by flow cytometry. The bar graph shows subpopulations of mono-, double-, and triple-positive CD4+ and CD8+ T cells. Two-way ANOVAs with Tukey’s multiple comparison test were used to compare vaccine groups against the same group adjuvanted with IL-33. Asterix color represents the corresponding cytokine groups. * = *p* < 0.05, ** *p* < 0.01, *** = *p* < 0.001, and **** = *p* < 0.0001. Values represent the mean responses in each group (*n* = 5) ± SEM.

To investigate the immunogenicity of co-formulated vaccine constructs groups of five mice were immunized 4 times at 3-week intervals with combinations of constructs (EXP1_PFN alone, EXP2 and ICP, TMP21 and UIS3, and all constructs together) with and without the molecular adjuvant pIL-33 all delivered in a single injection site. Splenocytes were collected for analysis of the cellular immune response using IFNγ ELISPOT and flow cytometry. All immunization groups saw an upwards trend in IFNγ ELISPOT responses with the addition of pIL-33 (Figure 3A). The functional profile of CD4+ (Figure 3B,D–F) and CD8+ (Figure 3C,G–I) T cells was analyzed by flow cytometry. In the mice immunized with EXP1_PFN alone, CD4+ T cells respond more highly to EXP1 and this phenotype is enhanced in the adjuvanted group (Figure 3D). By contrast, as seen in Figure 3G, CD8+ T cells respond more highly to PFN, and this phenotype is also enhanced in the adjuvanted group. In mice immunized with EXP2 + ICP, CD4+ T cells responded quite similarly to EXP2 and ICP; however, in the adjuvanted group the EXP2 response was preferentially increased (Figure 3E). CD8+ T cells responded more highly to EXP2; however, this phenotype was not recapitulated in the adjuvant group (Figure 3H). In the mice immunized with TMP21 + UIS3, CD4+ T cells responded more highly to UIS3 (Figure 3F), a phenotype that did not change with the addition of pIL-33, whereas CD8+ T cells responded more highly to TMP21 (Figure 3I). As expected, ELISPOT and flow cytometry responses in mice immunized with the empty vector control, pVax, or pIL-33 alone were negligible (Figure 3A–C). 

### 3.4. Py LS Antigen Vaccination Elicits a Robust and Polyfunctional Antigen-Specific T Cell Response in the Liver Which Is Enhanced with the Addition of Plasmid IL-33

**Figure 4:**
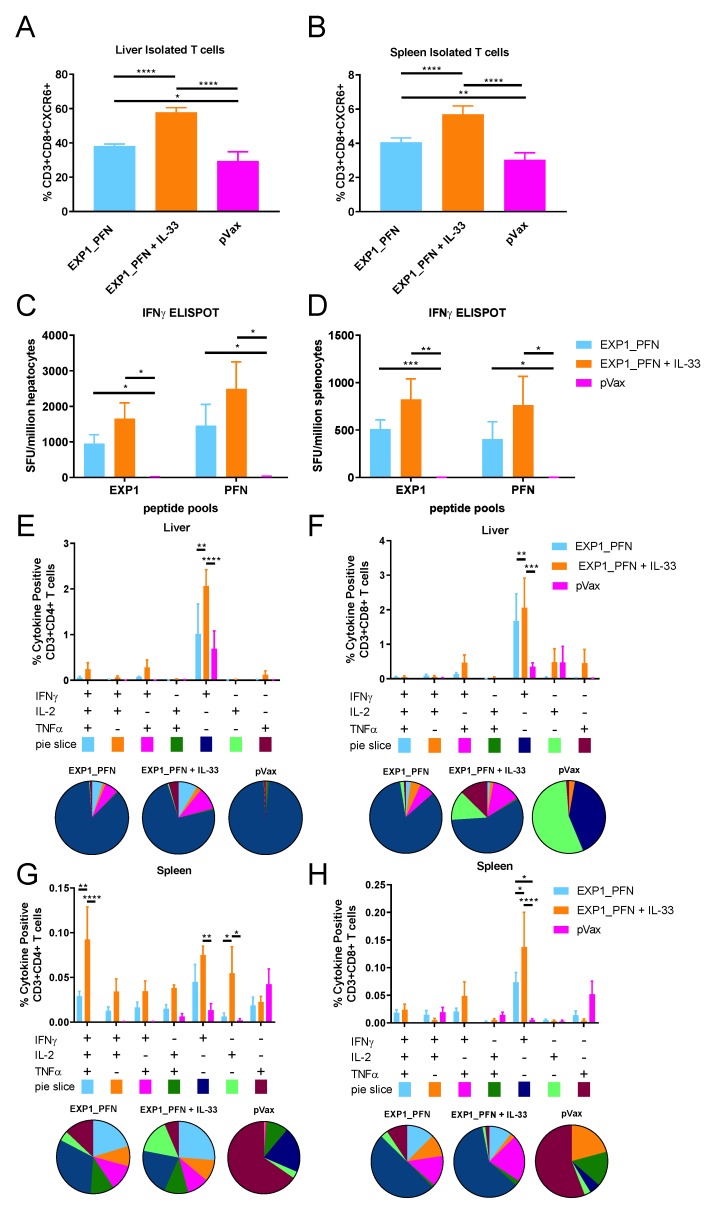
Synthetic DNA vaccines drive antigen-specific liver resident T cells. Mice were immunized 3 times at 3-week intervals with the EXP1_PFN vaccine construct with and without plasmid IL-33. Lymphocytes were isolated from liver and spleen 1 week after the final immunization. (**A**,**B**) The proportion of liver resident/homing CD8+ T cells. Lymphocytes from liver (**A**) or spleen (**B**) were stained for extracellular CXCR6 and analyzed by flow cytometry. Paired t-tests were used to compare the percent CXCR6 positivity on CD3+CD8+ T cells across vaccine groups and against the pVax control. (**C**,**D**) The *Py* antigen-specific cellular immune response in liver (**C**) and spleen (**D**) measured by IFNγ ELISPOT. Cells were stimulated for 18 h with peptide pools encompassing the entire protein. T-tests were performed to compare IFNγ production across vaccination groups and against the pVax control. The *Py* antigen-specific cytokine production profile of CD4+ (**E**,**G**) and CD8+ (**F**,**H**) T cells from the liver (**E**,**F**) and spleen (**G**,**H**). Cells were stimulated with pooled peptides for 6 h, stained for intracellular production of IFNγ, TNFα, and IL-2, and then analyzed by flow cytometry. The bar graph shows subpopulations of mono-, double-, and triple-positive CD4+ and CD8+ T cells. Two-way ANOVAs with Tukey’s multiple comparison test were used to compare cytokine production across vaccination groups and against the pVax control. * = *p* < 0.05, ** *p* < 0.01, *** = *p* < 0.001, and **** = *p* < 0.0001. The pie chart shows the proportion of each cytokine subpopulation. Values represent the mean responses in each group (*n* = 5) ± SEM.

The functionality of liver-localized antigen-specific T cells was investigated using the EXP1_PFN construct, as EXP1_PFN consistently produced the most robust T cell response. Mice were immunized 3 times at 3-week intervals with the EXP1_PFN construct with and without pIL-33. One week after the final immunization lymphocytes were isolated from both liver and spleen of immunized mice and their phenotype and functional capacity was measured using flow cytometry and ELISPOT assays. The percentage of CD8+ cells in the liver expressing CXCR6, a chemokine receptor important for trafficking to the liver [23], was significantly increased from 40% without co-delivery of pIL-33 to 60% with the addition of pIL-33 (Figure 4A) while the expression of CXCR6 on CD8+ cells in the spleen was 10-fold lower (Figure 4B) than that on CD8+ cells in the liver. This suggests an improvement of trafficking to the liver with the addition of pIL-33. As seen previously, the addition of pIL-33 increased antigen-specific IFNγ ELISPOT responses in the spleen (Figure 4D) and interestingly, also in the liver (Figure 4C). Further investigation of the functionality of the liver-localized T cells using flow cytometry revealed that pIL-33 not only increased the percentage of IFNγ+TNFα+IL-2+ (triple positive), IFNγ+, and IL-2+ CD4+ cells (Figure 4G) and IFNγ+ CD8+ (Figure 4H) cells from the spleen, but also increased the percentage of IFNγ+ CD4+ (Figure 4E) and IFNγ+CD8+ (Figure 4F) cells from the liver.

### 3.5. Vaccine Delivered with and without Adjuvant Elicits Antigen-Specific Antibody Responses

**Figure 5:**
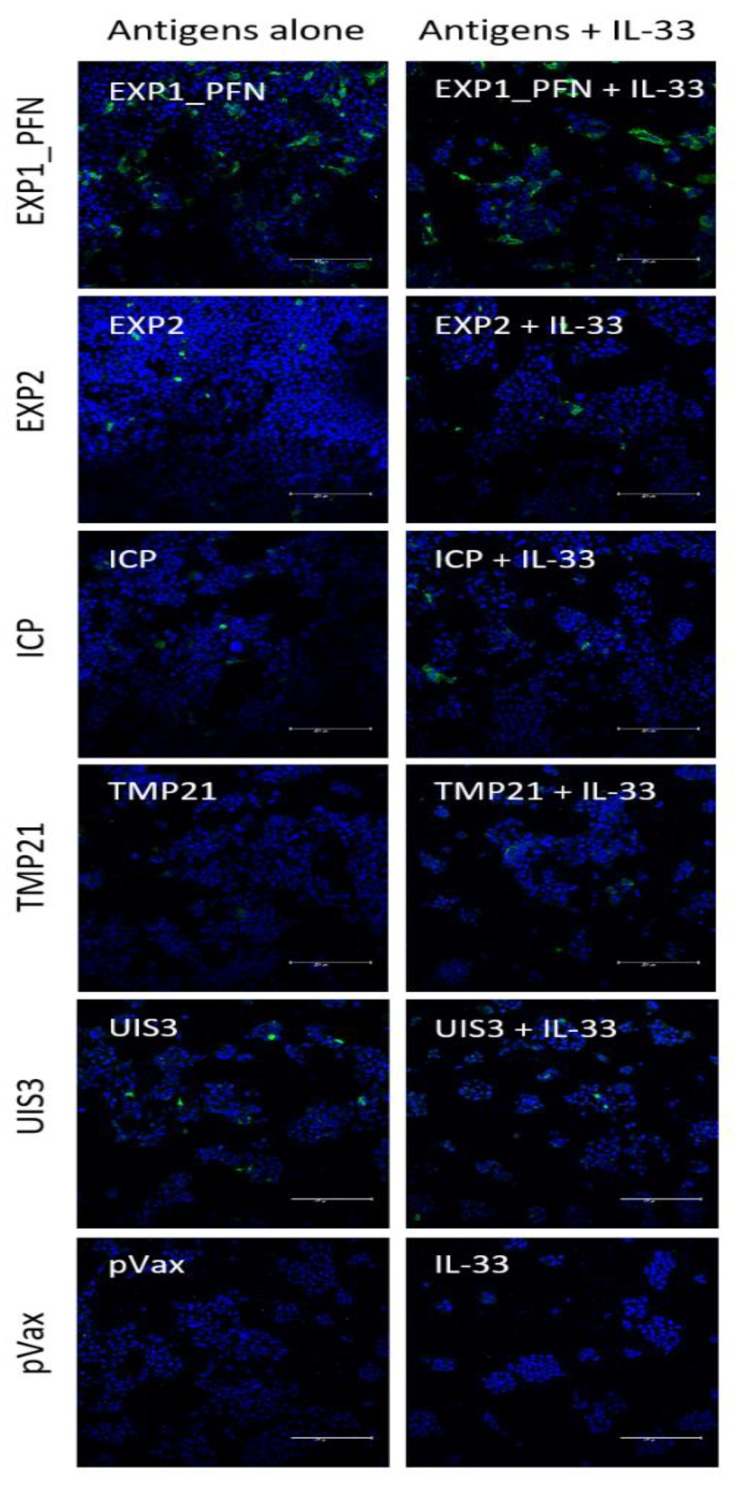
Antibodies elicited by *Py* DNA vaccines in mice. Hepa1–6 cells were transfected with the DNA vaccine construct listed on the left. Cells were then probed with pooled mouse post-immune sera collected 1 week after the last immunization. An anti-mouse-IgG-AF488 was used as a secondary antibody to detect the presence of anti-*Py* antigen antibodies. DAPI staining shows cell nuclei. White text in the top left corner of each field indicates post-immune sera vaccine group.

Vaccine-induced antibody production was assayed by immunofluorescence. Hepa1–6 cells were transfected with each DNA vaccine construct. Two days post transfection the cells were probed with post-immune sera from mice immunized with the respective DNA construct (with or without pIL-33) and probed with a fluorescently tagged secondary antibody. Mice immunized with EXP1_PFN showed the most positive staining indicating the highest antibody response. EXP2, ICP, and UIS3 showed intermediate antibody levels and TMP21 showed the lowest levels of antibody induction (Figure 5). The addition of pIL-33 did not appear to alter the amount of detectable antibody binding (Figure 5). However, we cannot conclude whether or not this antibody response is important for the observed immunity, as it is unknown whether these *Plasmodium* proteins would be available for antibody binding in a physiologically relevant context. It is important to note that while this assay shows the presence of antibodies elicited by vaccination, it does not provide quantitative information on antibody level or titer.

### 3.6. Plasmodium LS synDNA Vaccine Is Protective against Malaria Infection in a Mouse Model

**Figure 6:**
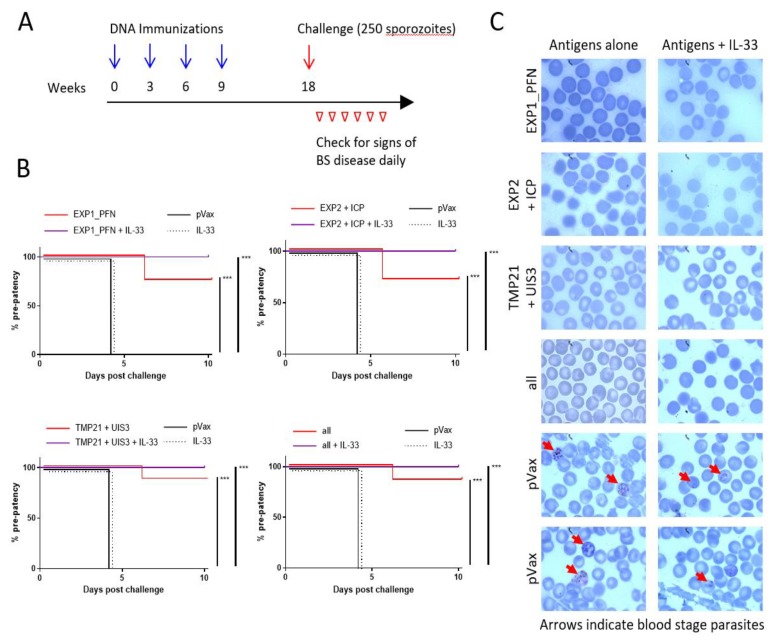
DNA vaccine expressing *Py* LS antigens provides protection from blood-stage disease after sporozoite challenge. (**A**) Vaccine and challenge timeline. BALB/cJ mice were immunized 4 times at 3-week intervals with the indicated vaccine co-formulations. Immunization groups were: EXP1_PFN with or without IL-33, EXP2 and ICP with or without IL-33, TMP21 and UIS3 with or without IL-33, and all vaccine constructs together with or without IL-33. Mice were then challenged by injection of 250 *P. yoelii* sporozoites. Blood smears were examined daily for signs of blood-stage disease. (**B**) Survival curves showing protection from evidence of blood stage parasites. Log-rank tests were used to compare groups and *p*-values less than the Bonferroni-corrected threshold are indicated. *** = *p* < 0.001. (**C**) Example blood smears from each group. Red arrows indicate blood stage parasites. All antigen immunized groups with and without IL-33 were *n* = 7 or 8. pVax and IL-33 groups were *n* = 5. See Supplementary Table S1 for more details.

To assess the ability of these DNA vaccine constructs expressing LS malaria antigens to protect against malaria, groups of 7–8 mice were immunized 4 times at 3 week intervals with combinations of constructs (EXP1_PFN alone, EXP2 and ICP, TMP21 and UIS3, and all constructs together) with and without the molecular adjuvant pIL-33, and then challenged 9 weeks later with intravenous delivery of 250 infectious *P. yoelii* sporozoites. Blood was collected daily following sporozoite injection for blood smears to check for the presence of blood stage parasites, i.e., patency (Figure 6A). All empty vector and pIL-33 alone immunized animals had visible blood stage parasites 4 days after challenge. In total, 71%–88% of animals immunized with DNA vaccine delivered without adjuvant were completely protected from patency, with parasitemia in the few animals who were not protected being delayed 1.5–2 full days. In total, 100% of animals immunized with any combination of constructs in addition to pIL-33 were completely protected from blood stage parasitemia indicating sterile protection in these groups (Figure 6B and Supplementary Table S1). Examples of the blood smears from each group showing the presence of blood stage parasites only in the pVax and pIL-33 alone groups are shown in Figure 6C.

## 4. Discussion

Pre-erythrocytic subunit vaccines in clinical development have shown limited success in clinical trials. Several studies have reported that T cell immunity to LS antigens contributes to protective immunity [14,15,23,24,25], suggesting that a combination vaccine approach targeting multiple life-cycle stages of the *Plasmodium* parasite may be important. Synthetic DNA delivered by adaptive electroporation (EP) is a particularly attractive vaccine platform for targeting LS antigens because of its ability to induce robust CD8+ T cell responses. In clinical studies, synDNA vaccines delivered with EP have proven highly effective in small and large animal models of infectious disease and cancer, and have demonstrated the ability to drive a tissue infiltrating population of antigen-specific CD8 T cells [47]. In recent years, DNA vaccines for HPV [47], HIV [58,59] Zika [45], and Ebola [60] among others have moved into human clinical trials. Here, we present a novel approach using synthetic DNA vaccination with LS antigens studied with and without the plasmid encoded molecular adjuvant IL-33 which together drive antigen-specific liver associated CD8+ T cells. This strategy achieved impressive protection from blood-stage disease in a virulent *Plasmodium* sporozoite challenge in the mouse model. 

The LS antigens EXP1, PFN, EXP2, ICP, TMP21, and UIS3 are expressed across *Plasmodia* making them enticing immune targets as they could provide protection from multiple species of *Plasmodia* that infect humans, as well as allow for study in murine models of disease. The synDNA vaccine platform has the advantage of ease of delivering cocktail vaccines targeting multiple antigens, as well as co-delivery of molecular adjuvants such as IL-33 to help shape the final immune response.

We designed synDNA vaccine cassettes expressing EXP1 and PFN, EXP2, ICP, TMP21 and UIS3 (Figure 1) and immunized mice with them individually, and in cocktails with each other and pIL-33. All vaccine constructs induced a strong cellular immune response, with EXP1, PFN, EXP2 and ICP inducing the highest IFNγ ELISPOT responses. TMP21 and UIS3 induced IFNγ ELISPOT responses were somewhat lower, but readily detectable (Figure 2). Intracellular cytokine staining revealed that while some antigens such as EXP1 and ICP induced a largely CD4+ T cell response, some such as PFN induced a CD8+ response, while others such as EXP2, TMP21, and UIS3 induced both CD4+ and CD8+ responses (Figure 2). When vaccine cocktails were delivered in combination with pIL-33, antigen-specific T cell responses increased compared to antigens alone (Figure 3).

Liver resident T cell responses are considered important for providing protective responses against LS antigens [23,24,25]. The proportion of CD8+ T cells expressing CXCR6 was increased in both the spleen and liver of animals immunized with EXP1_PFN plus pIL-33 compared to antigen alone. This was accompanied by a corresponding increase in the IFNγ production of antigen-specific CD4+ and CD8+ T cells in the liver of animals immunized with antigen plus pIL-33 (Figure 4). Together this suggests improved trafficking of vaccine-induced antigen-specific T cells to the liver.

To assess the impact of vaccine-induced immunity on *Plasmodium* infection, mice were immunized with different combinations of synDNA vaccine antigens with and without pIL-33 and challenged 9 weeks later with intravenous injection of *P. yoelii* sporozoites. Animals were then monitored daily for the development of blood-stage disease. In total, 71%–88% of animals immunized with vaccine antigen cocktails alone were protected from blood-stage disease, which increased to 100% protection in the animals immunized with LS antigens plus pIL-33 (Figure 6). This suggests that the increased CXCR6 expression and antigen-specific IFNγ production in the liver of animals immunized with pIL-33 and a likely corresponding increase in trafficking of vaccine-induced antigen-specific T cells to the liver might contribute to the improvement in protection from challenge. Further study is required to fully understand IL-33′s role in regulating these liver associated T cell responses.

While the longevity of the immune response induced by this synDNA vaccine targeting liver-stage *Plasmodium* antigens remains to be determined, long-lasting humoral and cellular immune responses induced by synDNA vaccines targeting other pathogens have recently been described. A synDNA vaccine expressing Ebola virus glycoprotein induced robust humoral and cellular immunity in cynomolgus macaques with minimal signs of contraction over a year. A booster immunization delivered at 1 year post first immunization resulted in a typical recall response with increased antigen-specific antibody titers and IFNγ ELISPOT responses. CD4+ effector memory (EM) and CD8+ effector memory RA (TEMRA) dominated the antigen-specific IFNγ and TNFα expressing T cells after the 1 year boost provided antigen re-exposure [60]. In a recent Phase I clinical trial, a synDNA vaccine expressing the pre-membrane and envelope (prME) proteins of Zika virus induced long-lasting humoral and cellular immune responses in humans. In total, 73% of participants who received the 6 mg dose of vaccine remained seropositive 48 weeks after their last immunization. Cellular immune responses also remained quite robust 48 weeks after the last vaccination [61]. Future studies will investigate the memory phenotype and protective potential of the long-term immune response to synDNA *Plasmodium* liver-stage antigens.

## 5. Conclusions

This study demonstrates that a synDNA vaccine targeting liver-stage *Plasmodium* antigens drives an antigen-specific liver-localized T cell population. Further, this study illustrates the potential efficacy of a synDNA vaccine platform targeting liver-stage proteins in providing protection from malaria infection in this model. We show that a synDNA vaccine targeting EXP1, PFN, EXP2, ICP, UIS3, and TMP21, in combination or alone, elicits a robust T cell response, as well as the production of antibodies against liver-stage malaria. The use of the molecular adjuvant pIL-33 increases the immune response to vaccine and protects 100% of animals from blood-stage disease after sporozoite challenge, where non-adjuvanted vaccine protects 70%–88% of animals from blood-stage disease. Potential contributors to this increased protection in the adjuvanted groups are the increase in antigen responsive liver associated T cells, or perhaps a dose-sparing effect of the adjuvant as previously reported for plasmid IL-12 [62,63], resulting in the IL-33 groups showing enhanced cytokine polyfunctionality. Additional dosing and other adjuvant studies remain important.

Additional focus on LS antigens as a component of a malaria vaccine is warranted, potentially in combination with sporozoite antigens such as CSP. RTS,S and R21 combinations might be particularly interesting [64,65,66]. In particular this seems a reasonable approach to improve on the current impact of such CSP targeting vaccines. This unique study contributes to a growing literature which collectively suggests that cell-mediated immunity in addition to antibodies should be further studied for design of multicomponent anti-malarial vaccines as well as further supporting the continued examination of liver-stage antigens as components of a prophylactic vaccine.

## Supplementary Materials

The following are available online at https://www.mdpi.com/2076-393X/8/1/21/s1. Table S1: Protection from blood stage parasitemia.
