# Supplementary material for: Synthetic DNA Vaccines Adjuvanted with pIL-33 Drive Liver-Localized T Cells and Provide Protection from Plasmodium Challenge in a Mouse Model
Source: Vaccines (Basel). 2020 Jan 10;8(1):21. doi: 10.3390/vaccines8010021 (PMC7157753; doi:10.3390/vaccines8010021)
Supplement: Supplementary file 1 [file vaccines-08-00021-s001.pdf]

vaccines

Table S1. Protection from blood stage parasitemia.

Construct	Immunization Intervals in Weeks	IV Challenge dose (days after Last Immunization)	Protected [+]/ Challenged	Average BS Pre-Patency in Days after IV Challenge for Infected Mice
EXP1_PFN	0, 3, 6, 9	250 (63)	6/8	d. 6 (for two mice)
**EXP1_PFN + IL-33**	**0, 3, 6, 9**	**250 (63)**	**7/7**	**-**
EXP2 + ICP	0, 3, 6, 9	250 (63)	5/7	d. 5.5 (for two mice)
**EXP2 + ICP + IL-33**	**0, 3, 6, 9**	**250 (63)**	**8/8**	**-**
TMP21 + UIS3	0, 3, 6, 9	250 (63)	7/8	d. 6 (for one mouse)
**TMP21 + UIS3 + IL-33**	**0, 3, 6, 9**	**250 (63)**	**8/8**	**-**
all	0, 3, 6, 9	250 (63)	6/7	d. 6 (for one mouse)
**all + IL-33**	**0, 3, 6, 9**	**250 (63)**	**7/7**	**-**
pVax	0, 3, 6, 9	250 (63)	0/5	d. 4 (for all mice)
IL-33	0, 3, 6, 9	250 (63)	0/5	d. 4 (for all mice)

+ Protection is defined as the complete absence of any BS parasites on daily blood smears for at least 10 days post challenge.
